# Using mHealth to provide sexual and reproductive health services to young people in rural Ghana: health care providers’ perspectives

**DOI:** 10.1093/heapol/czaf071

**Published:** 2025-11-13

**Authors:** Alexander S Laar, Melissa L Harris, Clare Thomson, Deborah Loxton

**Affiliations:** The University of Newcastle, Australia; School of Public Health and Medicine, Centre for Women’s Health Research, Faculty of Health and Medicine, Hunter Medical Research Institute, Callaghan, New South Wales 2308, Australia; The University of Newcastle, Australia; School of Public Health and Medicine, Centre for Women’s Health Research, Faculty of Health and Medicine, Hunter Medical Research Institute, Callaghan, New South Wales 2308, Australia; The University of Newcastle, Australia; School of Public Health and Medicine, Centre for Women’s Health Research, Faculty of Health and Medicine, Hunter Medical Research Institute, Callaghan, New South Wales 2308, Australia; The University of Newcastle, Australia; School of Public Health and Medicine, Centre for Women’s Health Research, Faculty of Health and Medicine, Hunter Medical Research Institute, Callaghan, New South Wales 2308, Australia

**Keywords:** mHealth platforms, sexual and reproductive health, information and services, young people, healthcare professionals, rural Ghana

## Abstract

Mobile health (mHealth) technologies are increasingly being used in innovative ways to overcome traditional barriers to the provision of, and access to, sexual and reproductive health (SRH) services among young people in rural low-and-middle income countries (LMICs). In rural Ghana, mHealth platforms are now being implemented by health care providers (HCPs) to improve access to SRH information for young people. However, the actual use of these platforms from the perspective of HCPs has not yet been explored. This study investigated HCPs’ perspectives on the availability of mHealth platforms in rural Ghana and the perceived benefits of using such platforms to provide SRH information and services to rural dwelling young people. A qualitative exploratory study using semi-structured interviews was conducted with a convenience sample of 20 HCPs across three rural regions of Ghana. Participants were recruited using the snowballing method between May and August 2021. Interviews were audio recorded via Zoom with participants’ consent. The data were transcribed verbatim and thematically analysed. All participants had experience providing mHealth-based SRH information and services to young people in rural Ghana. The mobile platforms used included phone calls, text messages, voice messages, Facebook, WhatsApp, and Twitter. These platforms facilitated SRH education on contraception,Human immunodeficiency Virus (HIV), sexually transmissible infections, hygiene, and menstruation. HCPs reported several benefits of using mHealth, including ease and convenience, low cost, anonymity, privacy and confidentiality (especially in light of socio-cultural norms and religious beliefs), reduced healthcare delivery workload, and reduced pressure on limited health infrastructure. The findings suggest that innovative mHealth platforms have the potential to improve young people’s access to conventional SRH information and services in rural Ghana. Furthermore, the findings demonstrate the preferred and acceptable use of these platforms among users. The results highlight the acceptability and utility of mHealth, as well as the need for its wider adoption and integration. While the provision of SRH information and services through mHealth is promising, further research is needed to understand the barriers that affect access and delivery for young people in rural communities.

Key messages

- In rural areas of Ghana, young people often have limited or no access to conventional SRH information.
- In response to challenges of SRH information and services delivery in rural Ghana, a mHealth programme was developed to increase access to SRH education and counselling via mobile phones.
- Despite the implementation of such mobile phone-based platforms in rural Ghana, limited information exists on how these services are used. This study addresses that gap by exploring HCPs’ perspectives on using mHealth to improve SRH services for young people in rural Ghana.
- The study findings will assist policy makers to successfully implement mHealth in the Ghanaian rural health system to address conventional provision and access barriers to SRH information and services.

## Introduction

In rural areas of low-and-middle-income countries (LMICs), including Ghana, young people (aged 10–24 years), often have limited or no access to information on sexual and reproductive health (SRH) (Obiezu-Umeh et al. 2021). Access to and use of conventional SRH services remain low due to various factors, including socio-cultural norms, religious beliefs, lack of privacy and confidentiality, limited awareness, social stigma, provider bias, transportation challenges, and costs(Obiezu-Umeh et al. 2021). These barriers partly explain the high unmet need for contraception information in LMICs (Chandra-Mouli et al. 2013, 2014, Obiezu-Umeh et al. 2021). Such unmet needs are linked to high rates of unplanned pregnancies, sexually transmitted infections (STIs), including HIV, and unsafe abortions among young people (Chandra-Mouli et al. 2013, Obiezu-Umeh et al. 2021).

To overcome these health system barriers, the World Health Organization (WHO) recommends creating supportive social environments that make services more accessible, acceptable, equitable, appropriate, and effective for young people (WHO 2019, 2020). Mobile Health (mHealth) defined as the use of mobile wireless technologies for health is one evidence-based intervention, particularly for providing youth-friendly SRH services (WHO 2019). In response to challenges of SRH services delivery and access in rural LMICs, many reproductive health programmes are now adopting mHealth solutions (Gottschalk and Ortayli 2014; Chandra-Mouli et al. 2015, WHO 2020). mHealth commonly involves the use of mobile phones and other wireless technology in medical care, with mobile devices being the most frequently used tools for educating clients about preventive health services (WHO 2020). The popularity of mHealth is growing especially in underserved areas with large populations and widespread mobile phone use. Nonprofit organizations like mHealth Alliance advocate for its increased use in developing countries (WHO 2019, 2020).

Although mHealth and telehealth may overlap, they are not interchangeable. While telehealth includes the delivery of remote care via electronic and telecommunications technologies (including mobile devices), it is a broader concept (WHO 2019, 2020). In Ghana, an example of mHealth is the Sexual Health Education Plus (SHE+) programme developed by Savana Signatures, a nonprofit organization. This initiative aims to increase access to SRH education and counselling by connecting young people with health professionals via mobile phones. Despite, the implementation of such mHealth platforms in rural Ghana, limited information exists on how these services are used. Specifically, there is gap in understanding the types of services provided, the nature of information and services delivered (e.g., contraceptives), and whether users benefit from these mHealth innovations.

This study seeks to address this gap by exploring HCPs’ perspectives on using mobile phones to improve SRH services for young people in rural Ghana. The following research questions guide the study:

1. What mobile phone based platforms are used by HCPs to deliver SRH services to young people in rural Ghana?
2. What are the perceived benefits of these platforms?

Through in-depth interviews with HCPs across rural Ghana, this research aims to generate new insights into how mobile technology might improve SRH information and services access for rural youth.

## Materials and methods

### Study sites

The study was conducted with HCPs located in three rural regions of Ghana (Volta, Upper East, and Northern Regions) where the Savana Signatures mHealth SHE+ programme has been implemented for young people. The age of the population indicates that all the districts of the region have a young population, with the highest proportion of rural coverage (Novignon et al. 2012). These rural regions have limited healthcare infrastructure and health personnel and the distance to healthcare facilities is far above the WHO recommended distance of 5 km (Atuoye et al. 2015) thus creating inequalities in access to health services and information (Kruk et al. 2016).

### Study design

This study used an exploratory qualitative design with semi-structured, in-depth interviews conducted via Zoom with HCPs in the rural communities in Ghana. This research approach provided the opportunity for an in-depth exploration of HCP’s views about mHealth based SRH information and services for young people in rural Ghana. The participants were recruited using convenience sampling (Sadler et al. 2010) and the snowball method. The qualitative interview tool was developed in English (the official language of Ghana). The semi-structured in-depth interviews consisted of a dialogue between the researcher and participant, guided by the interview schedule and supplemented by follow-up questions and probes. A qualitative research approach was chosen as it provides participants with the opportunity to express themselves and share their varied range of experiences in an in-depth and narrative manner (Crinson et al. 2016).

### Data collection procedure

**Figure 1.**
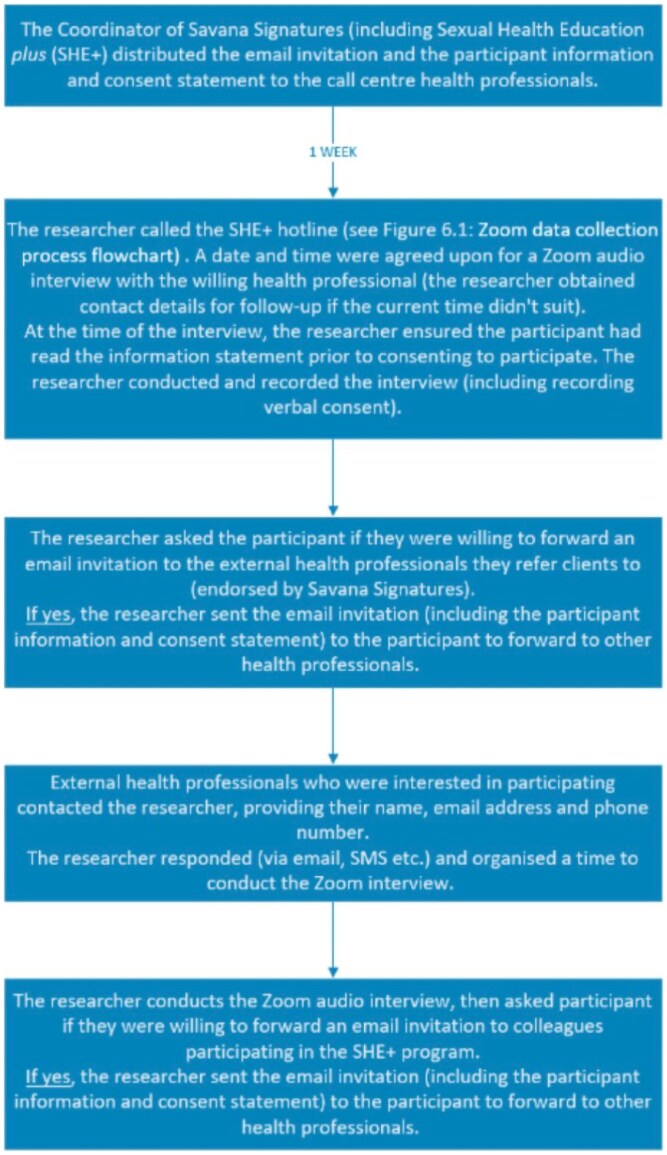
Study recruitment flow chart.

The study was conducted by the principal researcher between May and August 2021. The study tools were pre-tested in a similar environment with five HCPs (three males and two females) who had experiences of providing mHealth SRH services in rural Ghana but were not included in the main sample. Pilot participant responses were used to make the necessary changes to strengthen the interview guide. The participant pool was comprised of Savana Signatures Sexual Health Education Plus (SHE+) call centre staff and external HCPs, who were first approached by the management of Savana Signatures. Savana Signatures distributed the interview materials comprising of a flyer, letter of invitation, and information statement via email. The external HCPs being invited to take part in this study were health providers of mobile-phone-based SRH information and services in health facilities in rural Ghana. The health professionals included health care workers in the community, such as medical/frontline health workers (doctors, nurses, midwives) and allied health workers (health professionals who are not clinicians). These Health professionals were those who had experience disseminating in- and out-of-facility SRH information and services to young people in rural areas via mobile phone platforms informally. Health providers who were not delivering mobile phone-based SRH information and services were not eligible to participate. The letter of invitation provided information regarding the recruitment procedures, the researchers involved, study requirements, confidentiality provisions and potential risks related to study participation. Contact details of the researcher and principal supervisor were provided for participant queries. The study recruitment flow process is detailed in the flow chart in Fig. 1.

**Figure 2.**
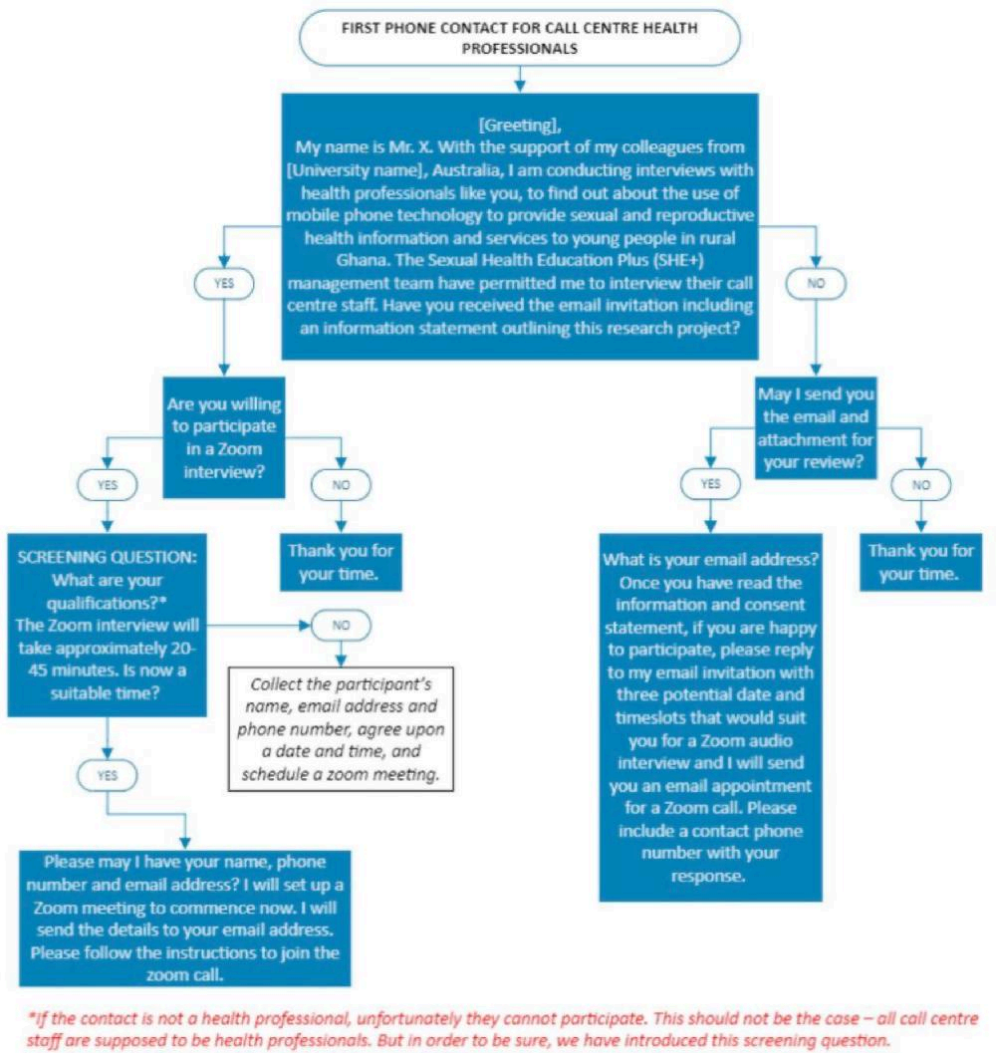
Zoom data collection process flow chart.

Participants who agreed to take part in the study were sent a Zoom link via email. Participants provided oral informed consent prior to conducting the interview. Initial participants passed on an invitation to other potential colleague HCPs. The study data collection process is detailed in the flow chart in Fig. 2.

Recruitment continued until the point of data saturation, where additional interviews no longer resulted in additional information (Mason 2010, Fusch and Ness 2015). The length of each interview ranged between 25 and 65 min. In all, 28 potential participants were initially approached and 20 were recruited using the convenience and snowball methods (Sadler et al. 2010).

### Data analysis

Prior to data analysis, the audio recordings were first transcribed verbatim and the transcripts read through for accuracy and familiarization (Halcomb and Davidson 2006). This process allowed the researcher to get a better feel and understanding of the data. Data were analysed thematically following the approach outlined by Braun and Clarke (Braun and Clarke 2006, 2014). The coded data were proofread against the original audios to ensure accuracy and quality. Prior to the analysis stage, initial ideas were noted in a memo created in an NVivo journal (Braun and Clarke 2006, Halcomb and Davidson 2006). All transcripts were coded using NVivo qualitative data management software (version 12). The thematic data analysis helped to generate specific broad themes and sub-themes. The study results were presented under these themes, supported by quotes of participants views.

### Rigour and reflexivity

This study demonstrated rigour through several methodological strategies. Reflexivity was maintained throughout the data collection and analysis process. The researcher kept detailed field notes capturing their thoughts and feelings about each interview, including reflections on the researcher-participant relationship and the influence of the researcher’s personal perspectives. This documentation enabled ongoing critical reflection on potential biases and their impact on the research process and interpretation. Confidentiality and anonymity were rigorously upheld. All transcripts were de-identified by assigning letter codes to participants, and all references to specific places or personal details were replaced with generic descriptors. Informed consent was obtained verbally prior to each interview, and participants were reminded of their right to decline questions or withdraw from the study at any time. To enhance the credibility and reliability of the findings, multiple researchers were involved in the data analysis and interpretation stages. Regular team discussions were held to compare perspectives, clarify interpretations, and strengthen the validity of the thematic analysis. The study also ensured diversity in the sample by using both convenience and snowball sampling techniques, which helped recruit HCPs with a broad range of experiences in delivering mHealth-based SRH services in rural Ghana. These efforts contributed to the richness and transferability of the findings. Finally, transparency in reporting the research design, data collection, and analysis enhances the trustworthiness of the study. These measures collectively ensure that the findings are dependable and relevant for informing policy, practice, and future research on mHealth interventions in rural healthcare systems.

## Results

### Participants

A total of 20 HCPs participated in this study. All the participants were health care providers working in rural health facilities. Among the participants, two were from Volta region and nine each were from the Northern and Upper East Regions. Thirteen participants were female and seven were male. Participant ages ranged from 25 to 42 years., Thirteen participants were married, and seven were single. Seventeen participants identified as Christians. All participants were highly educated. The sample included diverse healthcare roles: three medical doctors, seven nurses, two midwives, one community health nurse, four public health nurses, two call centre counsellors, and one psychologist. The participants’ healthcare delivery experience ranged from 2 to 16 years.

### Health care providers views on mHealth for sexual and reproductive health information and services

All HCPs had an understanding of using mHealth for the delivery of SRH information and services to young people in rural communities in Ghana. HCPs used different types of mHealth platforms including phone calls, WhatsApp, text messages, voice messages, Facebook, and Twitter. This is shown in Table 1.

**Table 1.** Types of mHealth platforms used for SRH information and services.

	Participants																				
mHealth platform	A	B	C	D	E	F	G	H	I	J	K	L	M	N	O	P	Q	R	S	T	Total
Phone calls	x	x		x	x	x	x	x	x	x	x	x	x	x	x	x	x	x	x	x	19/20
Facebook	x		x	x																	3/20
Short message service (SMS)/text messages	x	x	x			x		x	x	x			x	x		x	x				11/20
WhatsApp	x	x	x	x		x		x	x	x			x			x	x	x	x	x	14/20
Twitter			x																		1/20
Voice messages	x																				1/20

Participants indicated that they used mobile phones platforms for the delivery of education and counselling on contraception, menstruation hygiene, STIs and HIV prevention to young people. Two midwives indicated that use of the phone for SRH services delivery is done informally alongside the provision of face-to-face services.

…what I know is that most of the health staff in rural communities, especially in sexual and reproductive health units, are now using the mobile phone platforms informally for the education and counselling of young people on their sexual and reproductive health…we use both the phone and the face-to-face services in our facilities in rural communities…—Participant R, Senior Staff Nurse.

…most of the HCPs use the mobile phones to contact health for the delivery of SRH information and education in the communities alongside the in-person services…—Participant P, Midwife.

The HCPs indicated that they used different mHealth platforms to provide education and counselling on SRH information and services on contraception, menstruation hygiene, STIs and HIV prevention education to young people.

…health providers use the mobile phone to educate and counsel young people on their sexual health issues on contraceptives, avoid getting unintended pregnancy…condoms use, avoidance of STIs, menstruation hygiene…—Participant P, Midwife.

Participants also noted that the use of mobile phone platforms for the delivery of SRH information and services to young people depended on the needs of the users. This included the needs of specific rural areas in the community. A public health nurse said:

…in my community here, it’s the phone calls alone that we use…because the young people use the simple phones…they don’t have android phones…so we send the information and services depending on their needs at a particular time and place.—Participant G, Public Health Nurse.

All the participants emphasized that the mobile phone-based SRH services are delivered alongside the face-to-face services in their communities. They further indicated that they are only able to provide sexual health counselling services to young people when they are referred from Savana Signatures call centre staff.

… we use the mobile phone alongside the in-person services to address some of the conventional challenges among young people when they are referred to them from Savana Signatures call centre staff … after the first phone contact is established through Savana Signatures call centre staff, we are able to follow up on them or for them to contact us when there is a need— Participant G, Public Health Nurse.

### Preference for phone calls and text messaging

HCPs stated that phone calls and text messages were the most preferred platforms due to the affordability of basic mobile phones. Social media apps like WhatsApp, Facebook and Twitter were less commonly used by vulnerable young people due to the cost of smartphones and data.

…young people often contact us via phone call and text messaging because they can use basic phones….apps like Facebook and WhatsApp need smartphones, which are expensive …—Participant G, Public Health Nurse.

Phone calls were also preferred due to the lower literacy levels among some youth:

…I think the phone calls works well because they don’t require writing or reading skills…—Participant B, Health Counsellor.

…not everyone can write or read, and some youth lack the technical skills for text-based platforms…. … illiteracy is high in in our rural communities—Participant P, Midwife.

However, airtime costs made some youth call and hang up, expecting HCPs to call back. Conversely, text messaging was perceived as affordable and reliable even in areas with poor network coverage:

… sending text messages is cheaper and works well in areas with poor network connectivity …—Participant B, Health Counsellor.

Some HCPs preferred platforms like Facebook or WhatsApp for group outreach:

…some of us prefer text platforms like WhatsApp and Facebook because they allow us to reach large groups of young people with SRH information at once …—Participant J, Principal Medical Officer.

Participants emphasized that not all youth owned mobile phones or had awareness of mHealth SRH services:

…we use mobile phones and in-person services….but many youths still depend on face-to-face services because they lack mobile phones or information—Participant Q, Senior Staff Nurse.

… some HCPs aren’t aware of mHealth services …there is no public education on it… Participant P, Midwife.

### Perceived benefits of mHealth for accessing sexual and reproductive health services

All participants perceived diverse advantages associated with the use of mHealth platforms for the provision of SRH services to young people, including ease and convenience, low cost of services, privacy and confidentiality, reduction in access stress as well as being user-friendly. Participants perceived that these factors helped overcome a wide range of conventional access barriers, particularly those associated with socio-cultural norms and religious beliefs.

#### Ease and convenience of accessing mHealth sexual and reproductive health services

All participants perceived that young people using mobile platforms to access SRH information and services did so due to the convenience and ease of obtaining the services from their homes. This reduced the burden associated with accessing face-to-face services. Use of mHealth services also saved time by avoiding the hassle of long waiting times at the health facilitates.

…using the mobile phone planforms services is easier and convenient compared to the face-to-face consultations which require travelling to health facilities and as well as long waiting times to get the services from the comfort their homes…—Participant B, Health Counsellor.

…young people can contact health providers for the same sexual health information without the hassle of going to the health facility to queue for services…it eliminates the stress of waiting a long time for services as well as reducing workload health providers for seeing large groups of people… Participant Q, Senior Staff Nurse.

#### Privacy and confidentiality

The discrete nature of the platform’s services was perceived to increase young people’s confidence in using the SRH services, a level of privacy that could not be obtained during face-to-face encounters. Participants maintained that the remote use of the services also helped circumvent several community factors, especially those related to social and religious norms.

…using the mobile phone for services is more youth-friendly…the services meet young people’s privacy and confidential sexual health needs because of the anonymity it provides…—Participant C, Call Centre Counsellor.

The platform services are youth friendly…it makes the young people able to discuss their sensitive sexual health issues or problems with health providers openly without fear or shyness compared to the face-to-face encounter… Participant P, Midwife.

#### Reduction in sexual and reproductive health costs

Participants perceived that the use of mobile phone platforms for accessing SRH information and services are cost-effective for young people in rural communities. In the rural areas, health facilities are in the district capital towns which are often quite far from the communities. Accessing health facilities for SRH requires young people to travel long distances, which involves the cost of transportation and other miscellaneous expenses. The use of mobile phones helped eradicate or reduce the cost and stress associated with travelling to health facilities for SRH services compared to face-to-face health services.

The use of the mHealth platform for SRH services helps avoid stress and transport costs for health facility visitations in the district capital towns…—Participant G, Public Health Nurse.

#### Reduction in stress for waiting times for sexual and reproductive health services

HCPs also perceived that accessing SRH services remotely helped reduce the stress associated with waiting times at the health facilities.

…it reduces pressure on young people for joining long queues to see health providers at the health facility and the associated stress compared to the face-to-face services…—Participant L, Medical Officer.

#### Wide geographical coverage with sexual and reproductive health services

All participants reported that using mobile phones helped to reach young people across a wide geographical area. Some HCPs argued that using the mHealth platforms made it possible to reach large groups and individuals across broad remote and hard-to-reach areas, as compared to delivering conventional face-to-face services.

…using the platforms for delivering sexual health information made it easy to engage with large groups of young people and individuals with services tailored to their needs which is not possible to reach using conventional services…—Participant E, Midwifery Officer.

Participants also spoke about the swiftness of using mHealth platforms to reach several young people with a wide range of SRH services compared to the face-to-face consultation. The view of a staff nurse illustrated this point:

…using the phone helped delivered a wide range of sexual and reproductive health information faster to groups young people compared to the face-to-face services…—Participant I, Staff Nurse.

#### Reduction in workload stress for delivering sexual and reproductive health services

Participants reported a reduction of stress among HCPs in delivering SRH services remotely to young people. The reduction in consultation workload compared to conventional services was a key factor.

…using the mobile phone reduces workload stress on health providers for seeing less people…as compared to the face-to-face services…—Participant L, Medical Officer.

Some participants also associated the advantages of mHealth platforms for SRH services with the reduction in stress and pressure on the health facility infrastructure, with fewer clients visiting face-to-face services.

…using the mobile for services helps cuts down on a lot of out-patient department visitations that are not needed…It also reduces pressure on the facility infrastructure with less clients now visiting the health facility for consultations…—Participant N, Medical Doctor.

### Health care providers views on the adoption of sexual and reproductive health mHealth platforms in the rural health system

All participants maintained that mHealth services should be adopted in the rural health system to help realize its full benefits and for sustainability. Some also believed that the integration of mHealth SRH initiatives will require legislation or policy backing for successful implementation and sustainability, including the development of a national policy road map.

…to ensure a successful adoption and integration will require a legal framework for mHealth policy…I think the government needs to work with policy makers and implementers such as the ministry of health and Ghana health service to come out with a policy to facilitate a successful adoption and integration…—Participant N, Medical Doctor.

Some HCPs noted that the scale up of mHealth SRH initiatives for young people should not completely replace conventional SRH services but run concurrently in the health system as some diagnoses require physical examination.

…though the mobile phone SRH services has a lot of advantages it should not replace the conventional services going forward. The in-person services are also required in some instances where the technology cannot be used like checking of body temperature and some health issues can’t be diagnosed through using the phone only…—Participant Q, Senior Staff Nurse.

Notwithstanding the acceptability and benefits of novel mHealth platforms for SRH services among users in rural contexts, participants think that its integration, scale up and sustainability, will require affordable mobile phones and airtime to help lessen the financial burden on users.

You know when the cost of the mobile phone is made cheaper for young people, they can afford their own to use, that will make them independent… offering flexible payment term for vulnerable young people will help them acquire for to use mHealth platforms for sexual health information…—Participant N, Medical Doctor.

Participants indicated the need to empower HCPs and users for effective use of mobile phone-based platforms for SRH services.

…not all we the health providers and the users can perfectly use the mobile phone-based platforms for the exchange of sexual health information due to lack of or limited technical skills…the issue of technological skills has made the use of phone calls as the most common channel for services… if we are giving some training in this area it will help improve our skills…–Participant T, Public Health Nurse.

## Discussion

This study explored HCPs perspectives in using mHealth platforms and the perceived benefits of delivering SRH information and services to young people in rural Ghana. To the best of our knowledge, this is the first study in rural Ghana to examine HCPs views on mHealth-based SRH services for rural youth. The findings demonstrate high levels of interest among HCPs, who perceived multiple benefits for both providers and young people. These insights are critical for informing policy aimed at achieving universal access to SRH services. The study also provides guidance for SRH service providers on selecting effective mobile phone platforms for engaging young people and HCPs in rural settings.

HCPs reported using a range of mobile phone-based platforms including phone calls, text messages, voice messages, Facebook, WhatsApp, and Twitter to deliver SRH information and services. They highlighted the effectiveness these platforms in overcoming traditional access barriers associated with conventional SRH services, particularly in remote and underserved communities. These findings align with global literature demonstrating the utility of mHealth technologies in bridging gaps in SRH service access across LMICs (Ippoliti and L’Engle 2017, WHO 2019, 2020, Laar et al. 2022, Ochieng et al. 2022). The findings have shown that novel mHealth technology platforms initiatives have the potential for addressing multifaceted conventional SRH information provision and access barriers and challenges in the rural and remote communities of Ghana and LMICs due to the numerous perceived benefits (Ippoliti and L’Engle 2017, Laar et al. 2022, Ochieng et al. 2022 ). Globally, novel mHealth approaches have been leveraged for SRH services, especially among rural young population due to the benefits (World Health Organization 2019, 2020).

In this study, most participants perceived mobile phone calls and text messages to be the most preferred among young people, compared to other platforms (e.g. Facebook, WhatsApp, and Twitter). This is consistent with other studies conducted in rural LMICs (Memiah et al. 2022 , McCarthy et al. 2018, Meghari 2020). The reasons participants preferred text messages and phone calls platforms included the ability to use basic or simple low-cost mobile phones compared with platforms that required smart phones, and the ability to send and to receive messages to groups of people even under poor network conditions. This finding is consistent with a study in rural Nigeria which explored young people’s views on the opportunities and barriers for using the mHealth for SRH information and services (Akinfaderin-Agarau et al. 2012). According to the WHO, the use of digital mHealth platforms such as texting and phone calls are popular and increasingly used approach for the strengthening of the health system to address inequalities for use of SRH information and services among rural young population in LMICs (WHO 2019, 2020). Worldwide, mHealth technology platforms have been leveraged to provide youth friendly and acceptable SRH information and services outside the settings of the health facility in resource poor contexts (WHO 2019, 2020, Laar et al. 2022, Ochieng et al. 2022).

Phone calls and text messaging were reported as the most preferred platforms among young people. This preference is consistent with studies conducted in other rural LMIC contexts (Memiah et al. 2022, McCarthy et al. 2018, Meghari 2020). The popularity of these platforms was attributed to the widespread ownership of basic mobile phones and their affordability, especially in regions with poor internet connectivity. These observations align with research from rural Nigeria, which found similar constraints and opportunities related to mHealth use for SRH information and services (Akinfaderin-Agarau et al. 2012).

In the current study, smart phones were perceived as not affordable for young people particularly for those of low economic status. Low literacy levels among young people was cited as the perceived preference for phone calls, and literate young people for text messages, consistent with findings of a similar study conducted in rural Ghana (Peprah et al. 2020) and also corroborates with findings from rural LMICs (WHO 2020, Laar et al. 2022). As mobile phone calls were the most preferred platform among young people, it questions the practicality of scale-up and integration of mHealth for SRH services in rural health systems due to affordability for airtime. In our study, cost for airtime was perceived as a factor which influenced utilization of phone calls for SRH services among young people consistent with other research conducted in rural and remote LMICs (Vahdat et al. 2013, Laidlaw et al. 2017, Visser et al. 2020, Ybarra et al. 2020, Babatunde et al. 2021).To facilitate and guarantee effective use of mobile phones for SRH information and services especially among vulnerable young people, participants suggested implementation of cost reduction interventions by introducing low-cost phones on instalment payments and toll-free calls. Addressing financial issues is important for promoting ownership of phones and connection of services to permit independent use to help create a space for confidential service access to express their SRH needs with HCPs, without financial hardship or stigma (Babatunde et al. 2021) to facilitate utilization of services in rural healthcare system of Ghana and in other LMICs.

While text-based platforms services were preferred by some HCPs for disseminating SRH information to large groups, participants acknowledged limitations related to low literacy levels among rural youth. This highlights the need for tailored interventions that account for literacy and language preferences (Peprah et al. 2020). A research suggests that delivering SRH messages in local languages through text or voice platforms can significantly improve reach to SRH information and services (Babatunde et al. 2021).

In addition to improved accessibility, HCPs valued the anonymity and privacy afforded by mHealth platforms. These characteristics were seen as crucial for addressing stigma, provider bias, and socio-cultural taboos. Similar findings have been reported in other rural LMIC studies, where mHealth tools helped youth circumvent provider discrimination and community-level stigma and influences (Vahdat et al. 2013, Evelia et al. 2015, Blanc et al. 2016). Participants also noted logistical benefits of mHealth use, including reduced healthcare facility congestion, shorter wait times, and lower travel and consultation costs for clients. These factors contribute to improved equity in service provision and support the case for formalizing mHealth within Ghana’s rural health system. Participants suggest that its adoption should not replace the conventional services completely aligning with WHO recommendations (WHO 2020).

Despite these advantages, HCPs noted several barriers to scale-up and sustainability. These included limited public awareness, low digital literacy, and the cost of mobile devices and airtime. Community and school restrictions on phone use were also mentioned as obstacles. These findings underscore the need for coordinated efforts among policymakers, community leaders, and educational institutions.

All participants supported the adoption and integration of mHealth platforms into the rural healthcare system, while cautioning that mHealth should complement rather than replace in-person services since some clinical issues, such as physical examinations, cannot be addressed remotely. Participants advocated for national mHealth legislation and policy guidance to support implementation and ensure long-term sustainability. These recommendations are consistent with WHO guidance on integrating digital health into national systems (WHO 2020).

### Implications for policy and practice

The study findings highlight the perceived benefits of using novel mHealth platforms for delivering SRH information and services among young people in rural Ghana. These results are highly relevant for policy makers and implementers working towards the successful adoption and integration of mHealth platforms into Ghana’s rural health system. The findings also offer practical implications for scaling up of mHealth-based platforms to achieve universal access to SRH information and services as aligned with Sustainable Development Goals (SDGs) particularly SDG3 (Judd 2020).

For mHealth platforms to be effectively adopted and integrated into rural healthcare systems, a national policy framework and legislative backing is essential. Although Ghana has an mHealth framework, legislation supporting its formal adoption is lacking. This is partly due to limited research on the perspectives of policymakers. To address this gap, the government must prioritize the development of mHealth policies and legal instruments that guide implementation and promote sustainability. Effective integration of mHealth platforms requires a coordinated approach involving government institutions, healthcare stakeholders, community leaders, and NGOs. Government commitment is particularly crucial for facilitating scale-up, funding, and the establishment of clear policy directions.

Additionally, there is a need to consider affordability and accessibility in the rollout of mHealth initiatives. This includes providing low-cost mobile phones and flexible payment plans for vulnerable youth populations, as well as expanding toll-free services to eliminate cost-related barriers. These actions will help ensure independent and equitable access to SRH services through mHealth platforms.

Furthermore, investment in digital literacy and technology training for both HCPs and users is vital. Equipping users with the necessary skills will enhance platform utilization and encourage sustained engagement with SRH services. Public education campaigns should also be launched to raise awareness and acceptance of mHealth services, particularly in rural communities where misinformation and social stigma remain as obstacles.

By acting on these policy and practice implications, Ghana can strengthen its rural health system and move towards achieving universal access to SRH services for young people. These lessons are also applicable to other LMICs facing similar healthcare delivery challenges.

### Limitations and strengths

This study has several limitations. First, the findings may not be generalizable beyond rural Ghana due to the relatively small sample size, although the sample included diverse healthcare professionals. Second, interviews were conducted via Zoom rather than in-person, primarily due to the COVID-19 pandemic, which may have affected rapport-building and data richness. Third, the study focused solely on HCPs’ perspectives and did not include the voices of young people or community members, which could have provided more comprehensive insights into mHealth use for SRH services.

Additionally, although participants described using various mHealth platforms to engage with young people, the study was not designed to determine which platforms are most effective or appropriate in rural contexts. Future research should explore young people's preferences and experiences with different mHealth platforms to identify the most impactful tools for SRH service delivery. Despite these limitations, the study has important strengths. It provides valuable, context-specific insights into how HCPs are using mHealth platforms to address long-standing conventional access barriers to SRH services in rural Ghana. By capturing diverse professional perspectives, the study contributes to the limited but growing evidence base on the practical implementation and benefits of mHealth interventions in LMICs.

The findings offer useful guidance for stakeholders aiming to strengthen rural health systems through digital innovations. Including young people’s voices in future research will further enhance our understanding of the suitability, acceptability, and effectiveness of mHealth platforms in similar settings.

### Rigour and reflexivity

This study demonstrated rigour through several methodological strategies. Reflexivity was maintained throughout the data collection and analysis process. The researcher kept detailed field notes capturing their thoughts and feelings about each interview, including reflections on the researcher-participant relationship and the influence of the researcher's personal perspectives. This documentation enabled ongoing critical reflection on potential biases and their impact on the research process and interpretation.

Confidentiality and anonymity were rigorously upheld. All transcripts were de-identified by assigning letter codes to participants, and all references to specific places or personal details were replaced with generic descriptors. Informed consent was obtained verbally prior to each interview, and participants were reminded of their right to decline questions or withdraw from the study at any time. To enhance the credibility and reliability of the findings, multiple researchers were involved in the data analysis and interpretation stages. Regular team discussions were held to compare perspectives, clarify interpretations, and strengthen the validity of the thematic analysis.

The study also ensured diversity in the sample by using both convenience and snowball sampling techniques, which helped recruit HCPs with a broad range of experiences in delivering mHealth-based SRH services in rural Ghana. These efforts contributed to the richness and transferability of the findings.

Finally, transparency in reporting the research design, data collection, and analysis enhances the trustworthiness of the study. These measures collectively ensure that the findings are dependable and relevant for informing policy, practice, and future research on mHealth interventions in rural healthcare systems.

## Conclusion

The findings of this study demonstrate a high level of interest and perceived multiple benefits among HCPs for using mHealth platforms to deliver SRH information and services to young people in rural Ghana. Participants emphasized the urgent need for adopting innovative mHealth technologies within the rural health system to realize their full potential for achieving universal access to SRH services. The study highlights the acceptability and practical utility of mHealth, suggesting the need to scale up these services to complement and enhance traditional service delivery in underserved areas. The findings also point to the importance of monitoring and evaluating mHealth rollout, service uptake, and user outcomes to inform future programming. Further research is necessary to explore young people’s preferences and experiences with various mHealth platforms to identify the most appropriate tools for SRH service delivery. This insight will be critical for designing effective, youth-friendly digital health interventions that are both accessible and sustainable. In conclusion, this study offers crucial guidance for government agencies, stakeholders, and policymakers in Ghana to design and implement practical mHealth strategies that strengthen rural healthcare systems and promote universal SRH access among youth.

## Data Availability

The data for this study are available from the corresponding author on reasonable request.

## Reflexivity statement

The authors include three females and one male of different levels of research experience and seniority. Three of the authors are from Australia. The corresponding author is from Ghana, West Africa with extensive experience in public health and health economics. Three of the authors have deep experience contributing to policy development for health system improvement. Three of the authors have experience in research work in Lower-and Upper middle-income countries. All authors have specialized in various areas of public health, health economics and health policy research. All the authors have extensive experience conducting qualitative research field work in Lower-and Upper middle-income countries. This study has benefitted greatly from the deep expertise of the authors in policy research making in LMICs in the health sector.
